# Detection of divergent *Orthohantavirus tulaense* provides insight into wide host range and viral evolutionary patterns

**DOI:** 10.1038/s44298-024-00072-y

**Published:** 2024-12-04

**Authors:** Mert Erdin, Teemu Smura, Kursat Kenan Kalkan, Ortac Cetintas, Muhsin Cogal, Sercan Irmak, Ferhat Matur, Ceylan Polat, Tarja Sironen, Mustafa Sozen, Ibrahim Mehmet Ali Oktem

**Affiliations:** 1 Department of Virology, Medicum, Faculty of Medicine, University of Helsinki, Helsinki, Finland; 2 Department of Medical Microbiology, Institute of Health Sciences, Dokuz Eylul University, Izmir, Türkiye; 3 Department of Biology, Faculty of Science, Zonguldak Bulent Ecevit University, Zonguldak, Türkiye; 4 Science and Technology Application and Research Center, Balikesir University, Balikesir, Türkiye; 5 Department of Biology, Faculty of Science, Dokuz Eylul University, Izmir, Türkiye; 6 Virology Unit, Department of Medical Microbiology, Faculty of Medicine, Hacettepe University, Ankara, Türkiye; 7 Department of Basic Veterinary Sciences, Faculty of Veterinary Medicine, University of Helsinki, Helsinki, Finland; 8 Department of Medical Microbiology, Faculty of Medicine, Dokuz Eylul University, Izmir, Türkiye; 9 Izmir Biomedicine and Genome Institute, Dokuz Eylul University, Izmir, Türkiye

**Keywords:** Virology, Epidemiology

## Abstract

*Orthohantavirus tulaense* (TULV) is a member of the orthohantavirus genus and distributed in Europe and Asia. To shed light on TULV epidemiology and evolution, we trapped wild rodents from eastern Turkiye and found 15 TULV positive rodents. Sequencing and phylogenetic analyses confirmed the presence of diverse TULV strains. Global phylogenetic characterization suggested 5 distinct TULV lineages. Global phylogeographic reconstruction estimated different rooting times for each three segments, a potential ancestor location in Eastern Black Sea region, and strongly supported phylogeographic structure with 11 clusters. Dispersal velocity of TULV was estimated to be much faster than some other orthohantaviruses. Eastern Black Sea seemed to have lineages evolving faster and genetically closer to proto-Tula virus. Host switching estimates suggested potential switching events from *Microtus arvalis* to *M. obscurus* to *M. irani* with host-dependent sub-clustering within geographic clusters and suggested substantial evidence for no clear virus jumps from *M. arvalis* to *M. irani*.

## Introduction

Orthohantaviruses are globally distributed viruses, that use small mammals as reservoirs. Rodent-borne orthohantaviruses are divided into three major groups: Murid-borne, non-arvicolinae cricetidae-borne, and arvicolinae-borne^1^. Some of these rodent-borne orthohantaviruses can infect humans as accidental hosts by direct contact with rodents or indirectly through inhalation of rodent excreta, and cause disease in humans^2^. The human diseases caused by orthohantaviruses are nephropathia epidemica (NE), hemorrhagic fever with renal syndrome, and hantavirus cardiopulmonary syndrome, from the mildest to the most severe, respectively^3^. Their capability to cause severe diseases, potential for outbreaks in humans, and undetected diversity in the nature with unknown relevance to human health make orthohantaviruses a public health concern. Therefore, to prevent potential outbreaks and human cases, surveillance of wild rodents for orthohantaviruses as well as understanding their host range and circulation patterns is of great importance.

The distribution of orthohantaviruses is mostly limited by the distribution of their reservoir hosts. Traditionally, orthohantaviruses were separated into old world and new world orthohantaviruses based on the distribution of their reservoir hosts^4^. Currently, orthohantaviruses are considered highly diverse viruses with a broad host range including rodents, insectivores, and bats. The orthohantaviral phylogeny is not separated by intercontinental distribution but rather reservoir host-dependent separation^1,5^. For instance, *Oxbow orthohantavirus* reported from USA, is phylogenetically closer to other soricidae-borne orthohantaviruses distributed in Eurasia, suggesting host switch^6^.

Arvicoline-borne orthohantaviruses are hypothesized to be the geographic and evolutionary bridges between murid-borne and non-arvicolinae cricetidae-borne groups^1^. Most of the member species of arvicolinae-borne orthohantavirus group are non-pathogenic to humans with two known exceptions, *Orthohantavirus puumalaense* (PUUV) and potentially *Orthohantavirus tulaense* (TULV). PUUV is the causative agent for NE and it’s highly prevalent in European countries especially in Finland, Germany, Sweden, and Russia^2–4,7–9^. A recent report suggested that in addition to NE, PUUV may also cause a severe disease, acute respiratory distress syndrome, in rare cases^10^. TULV was initially reported to cause infection in an immunocompromised patient in Czech Republic^11^. Later, TULV infection was shown to cause clinical manifestations also in immunocompetent patients^12,13^. These rare cases and reports show TULV’s potential pathogenicity and emergence, and the need of more surveillance studies for the prevention of human cases.

The primary reservoir host of TULV is thought to be common vole (*Microtus arvalis*) since most of the earlier studies showed its high prevalence in these animals^14–17^. However, recent studies have showed wide host range of TULV in other vole species of genus *Microtus* and even in the members of other genera such as steppe vole (*Lagurus lagurus*, genus *Lagurus*, family *Arvicolinae*)^18^ and forest dormouse (*Dryomys nitedula*, genus *Dryomys*, family *Gliridae*)^19–22^. Whether these are recent cross-species spillovers or TULV has always had wide range of reservoir hosts, remains unknown. To shed light on host range and evolutionary dynamics of TULV, we performed a cross-sectional study to search for orthohantaviruses from rodents in the eastern part of Turkiye, and further evaluated phylogenetic, phylogeographic, and evolutionary characteristics.

## Material and Methods

### Ethical statement

Study design, animal handling and experimental procedures were approved by the Dokuz Eylul University Local Ethical Committee of Animal Experiments (No: 72/2016), and the General Directorate of Nature Conservation and National Parks, Ministry of Forestry and Water Affairs (No: 72784983-488.04-11467).

### Animal sampling

The distribution maps of potential orthohantavirus reservoir host species, *Apodemus* spp., *Microtus* spp., *Chionomys* spp., *Mus* spp., *Crocidura* spp., and *Cricetulus migratorius*, were extracted from IUCN in order to design animal sampling. We conducted animal sampling between July 2019 and August 2021. Trapping locations were chosen according to target rodent species ecology or their behavioral movements. Sherman-type live-traps were set in a total of 5224 sampling points in 75 sampling locations in 13 provinces. Ten meter intervals were set between each trap and the traps were placed considering previously characterized rodent distribution, habitat suitability of the target species, and rodent nests in the field. All trapping locations were GPS tracked. These GPS tracked sampling locations were different for each targeted rodent species (Supplemetary Fig. S1), and the numbers of sampling locations are as follows for each rodent species: *Apodemus* spp. 21, *Microtus* spp. 21, *Chionomys* spp. 11, *Cricetulus migratorius* 9, *Mus* spp. 6, and *Crocidura* spp. 7 locations. For *Crocidura migratorius* trapping, ‘pitfall’ method was used by burying plastic cups to the ground and checking the cups regularly for rodent fall. Trapped but non-targeted rodent species were released.

The trapped animals were sacrified by cervical luxation. We recorded body measurements for the phenotypic identification, and collected information on additional conditions such as pregnancy state, testicle abnomalies, splenomegaly, indications of internal macroparasite infections, and ectoparasites. We dissected the heart, liver, spleen, kidney and lung tissues, and stored them in RNA later at -80 °C freezer. Lung tissues were used for orthohantavirus screening, and other tissues stored for the additional pathogen screening for the future studies.

### Orthohantavirus screening

We used lung tissues for orthohantavirus molecular screening. We homogenized the tissues in TRIzol reagent, and later extracted total RNA by phenol-chloroform method followed by reverse transciption of total RNA for complementary DNA synthesis and pan-hantaviral PCR^23^. We confirmed that PCR positive samples were TULV by Sanger sequencing (OR453239-OR453253). We additionally extracted DNA from lung tissues and performed PCR targeting *cytochrome-b* (*cyt-b*) gene in mitocondrial DNA followed by Sanger sequencing for the molecular identification of the positive animals (OR475424-OR475438)^24^.

### Next generation sequencing

Orthohantavirus PCR-positive lung samples were pretreated in BSL-3 facility using slightly modified NetoVIR protocol^25^ for the viral enrichment. Glass beads were added to the samples followed by homogenization with Magnapure homogenizer for 1 min and 30 s, giving cooling down breaks in every 30 s in order to prevent viral protein degradation due to heat generation. We followed rest of the protocol as described previously without making additional changes^25^. In the end of the enrichment, we took the samples to TRIzol reagent and again performed RNA extraction by phenol-chloroform method. For the RNA library preparation, we used NEBNext rRNA depletion (human/mouse/rat) v2 kit and NEBNext ultra II RNA library prep kit. All libraries were sequenced with the Illumina NovaSeq 6000 system. We analyzed the raw data in LazyPipe to obtain the complete coding sequences (CDS) of each segment of TULV (OR453254-OR453268)^26,27^.

### Phylogenetic analysis

We first constructed maximum likelihood (ML) trees from partial nucleotide sequences of viral (L segment) and respective rodent *cyt-b* gene amplicons. We aligned the sequences by ClustalW in MEGA11^28^, and constructed trees in IQ-TREE 2^29^ by using ModelFinder^30^ to find best-fitted model for tree construction implemented in IQ-TREE 2. Later, we followed the same approach for the ML trees of each segment’s CDS to analyze the lineages. Additionally, we also checked amino acid substitutions in the encoded protein sequences specific to Middle-Eastern lineage from the alignments of each three viral proteins, and added these substitutions to constructed ML trees with their positions at the protein sequence. This lineage characterization required confirmation with bigger dataset. Because the highest number of available partial sequences belonged to S segment, we generated a new partial genome dataset with threshold of >500 nucleotide. We repeated ML tree construction as described above, and used this new partial dataset as both unrooted and rooted with *Orthohantavirus puumalaense* reference sequence. We used previously generated CDS datasets for each segment in bayesian inference, but tested levels of phylogenetic information embedded in datasets via likelihood mapping. We used 10,000 quartets for each segment and visualized in triangles for phylogenetic information classification which corresponds to that corners were informative and central section was uninformative.

### Continuous phylogeography reconstruction

We used BEAST v1.10.4^31^ for phylogeographic reconstruction in continuous space with CDS datasets of each segment, separately. We used same parameters for each analysis for consistency of estimates, and set parameters as follows: coalescent: Constant size as best-fit model, uncorrelated relaxed clock, general time reversible with gamma categories (4) and invariant sites (GTR + G4 + I) for the substitution model with codon partition as separate for each codon position, Markov chain Monte Carlo (MCMC) chain length to be 5 × 10^8^ and sampling in every 50,000. After confirming the performed analyses’ confidence were high enough (Effective sampling size (ESS) to be >200) in Tracer v1.7.2^32^, we performed post-burn-in subsampling of trees in every 500,000 to obtain 1000 trees in LogCombiner v1.10.4. Later, we extracted maximum clade credibility (MCC) tree of each datasets from this subsample 1000 trees in TreeAnnotater v1.10.4. We then assessed the confidence and phylogenetic correlation by measuring association index (AI) and parsimony scores (PS). To do that, we first converted our “.trees” file to readable format with beast2phy tool (https://github.com/nylander/beast2phy) in perl, and later used BaTS tool^33^ (https://github.com/lonelyjoeparker/befi-bats-gui) to obtain AI and PS values. In BaTS tool, we used parameters as states to be the number of each unique location and number of replicates (100) for each segment, separately. When *P* < 0.05 for both AI and PS, then the null hypothesis was rejected. Then, we continued for phylogeographic map reconstruction and we extracted spatiotemporal data by Spread3 for both MCC tree mapping and 80% highest posterior density regions from 1000 subsampled trees. Finally, we used Seraphim package^34,35^ in R v4.3.3 to extract 1000 trees regularly sampled from post-burn-in posterior distribution of continuous phylogeographic estimations (*n* = 10,000 due to 500,000 log sampling in 5 × 10^8^ MCMC length) followed by extraction of weigthed dispersal velocity and diffusion coefficiency statistics along with isolation-by-distance signals, which was introduced recently to this package^36^. As the last stage of continuous phylogepgraphy, we generated a dataset with S segment sequences of Adler and Middle-Eastern lineages for the Eastern Black Sea regional inference. We used following parameters: TN93 with invariant sites as best-fit site model, 3 sites codon partition was enabled, cauchy random relaxed walk with bivariate traits and 0.01 jitter size, uncorrelated relaxed clock, coalescent: constant size as best-fit model, 2.5 × 10^8^ MCMC chain length with sampling in every 25,000. We assessed the results, reconstructed phylogeography, annotated MCC tree, and estimated dispersal statistics as described above.

### Comparative and regional inference of evolution rates

In order to reduce sampling bias, we included sequences to this dataset only if they had all three segments available. These datasets had sequences representing Middle-Eastern, European, and Asian lineages. Analysis parameters were same as in previous section with two exceptions: geographic location traits weren’t used and we set MCMC chain length to 1 × 10^8^ with sampling in every 10,000. We evaluated our estimates as described in the previous section. We also generated another dataset which consisted of sequences of S segment from continuous phylogeography (all available CDS for S segment); however, we excluded sequences of Adler lineage from this dataset to assess the changes at the evolution rate. We conducted this analysis to test the importance of Adler lineage for the estimation. We used same parameters as in unbiased evolutionary inference except MCMC chain length which was set to 2.5 × 10^8^ with sampling in every 25,000.

Lastly, we used employed fixed local clock as molecular clock to estimate the age and evolution rates independently for the specified taxa(s). For this, we used exactly the same dataset as it was used in S segment continuous phylogeography. We assessed the independent evolution rate and tMRCA(s) of Middle Eastern and Adler lineages against rest of the dataset. We assigned specific taxas to sequences of Middle Eastern and Adler lineages and marked as monophyletic to estimate their age and evolution rate independently. This analysis also was set same as previously with changes at molecular clock type and MCMC chain length which was 3 × 10^8^ with sampling in every 10,000.

### Host switching inference

As a final step, we used same CDS datasets that we used in continuous phylogeography for host switching estimates. We performed this analysis only for S and M segments since L segment had TULV CDS only from three rodents species (*Microtus arvalis, M. obscurus, M. irani*). Because of estimate consistency, we set parameters as defined previously in phylogeography section. We first confirmed the confidence described as in previous section (ESS proportions, AI and PS calculations), and used unique host species as states in the calcuation of AI and PS with replicates. We used Spread3 to extract the ‘Bayesian Stochastic Search Variable Selection’ values for hypothesis evidential supports of host switching events. We used bayes factor scale for hypothesis confidence as follows: No support (<3), substantial (>3), strong (>10), very strong (>30), decisive (>100).

### Data analysis and visualization

Data analysis, processing, and visualization were performed in STATA and R version 4.3.1/R studio. Maps were constructed in ArcGIS Pro and QGIS softwares.

## Results

### Animal sampling

**Fig. 1:**
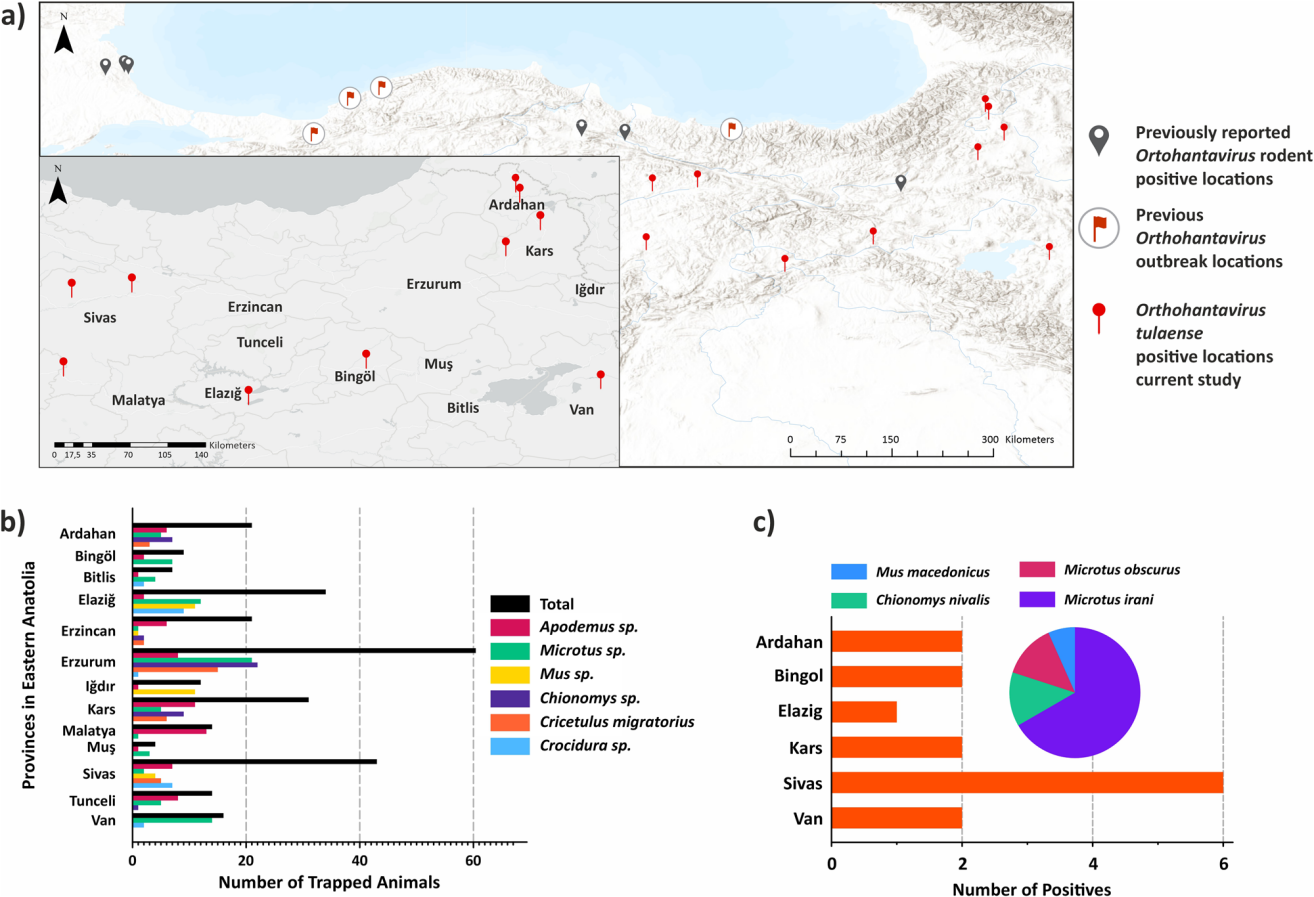
Orthohantavirus RNA was detected in 4 different rodent species captured from a total of 6 provinces. **a** The map represents previously reported studies for orthohantavirus detections in wild rodent populations, orthohantavirus outbreak regions and the locations with TULV detection in this study. **b** The numbers of captured rodents in each province. There were total of 293 rodent species trapped during the current study (see detailed all sampling locations, Supplementary Fig. S1, Supplemetary Table S1). **c** The number and host species distribution of orthohantavirus positive samples in each province. We detected 15 rodents from this collection as positive for orthohantavirus in molecular screening.

We trapped rodents from a total of 5224 trapping points in 75 sampling locations in 13 provinces, yielding a total of 293 rodent individuals (Supplementary Table S1). We detected orthohantaviral RNA in total of 15 rodents from 6 provinces (Fig. 1). None of these provinces have any previous records of orthohantavirus circulation in wild rodents. Together, orthohantavirus detections in the regions included in this study and the regions identified in the previous studies suggest that orthohantaviruses circulate in wild rodent populations of the northern and eastern parts of Turkiye (Fig. 1).

### Phylogeny of *Orthohantavirus tulaense*

For orthohantavirus species identification, we initially sequenced the amplicons of pan-hantavirus detection PCR with Sanger sequencing method. All 15 samples were TULV positive (OR453239-OR453253). These partial L segment TULV sequences clustered with previously reported TULV sequences from the same region^21^ (Supplementary Figure S2a). Molecular host species identification by *cyt-b* gene sequencing showed that *Microtus irani, Microtus obscurus, Chionomys nivalis*, and *Mus macedonicus* were positive for TULV (OR475424-OR475438) (Supplemetary Figure S2b). Of these, *Microtus obscurus* is known to be a reservoir host for TULV, but to our knowledge there are no previous detections of TULV in *M. irani*, *C. nivalis* and *M. macedonicus*.

**Fig. 2:**
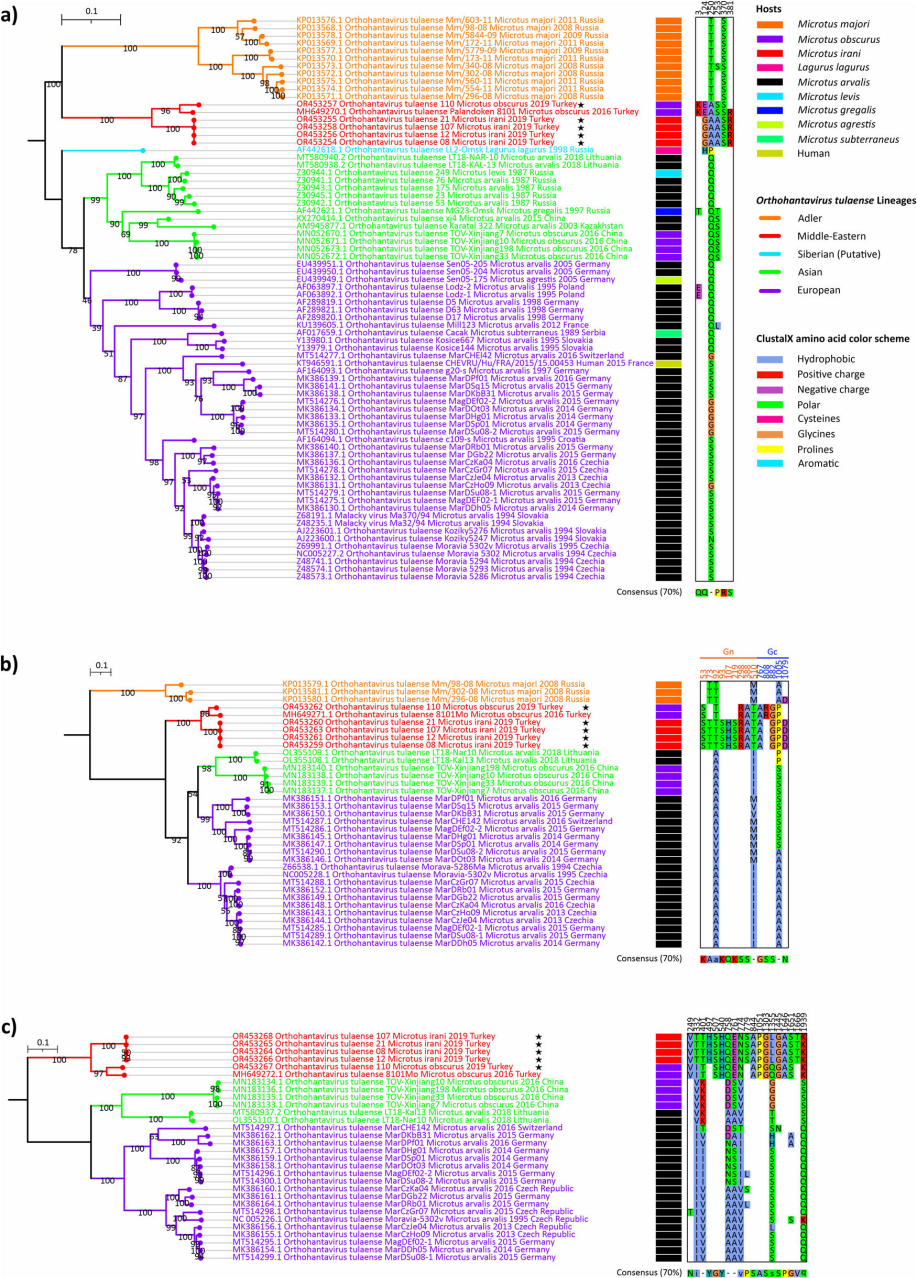
Maximum Likelihood trees based on CDS of all three TULV segments. The sequences obtained in this study are marked with black stars. Amino acid groups are colored according to ClustalX color scheme, and substitution positions are numbered according to open reading frame positions. **a** TULV S segment ML tree was constructed by TIM2 + F + I + R3 substitution model and 1000 ultrafast bootstrap replicates. **b** TULV M segment ML tree was constructed by GTR + F + I + R2 substitution model and 1000 ulstrafast bootstrap replicates. **c** TULV L segment ML tree was constructed by GTR + F + I + R3 substitution model and 1000 ultrafast bootstrap replicates.

Complete genome sequencing with next generation sequencing (NGS) was succesful from five samples (OR453254-OR453268). The phylogenetic analysis based on nucleotide sequences of the CDS of each segment with ML method suggested separation into five lineages, reflecting the geographic origins of sequences, named here as follows: Adler, Middle-Eastern, Siberian (putative), Asian, and European lineages (Fig. 2). Of these, the Siberian lineage is represented by a single S segment sequence and should therefore be considered as a putative lineage. The S segment ML tree with the highest number of available CDSs suggested these 5 lineages (Fig. 2a), but due to the lack of sequence data (L segment of Adler and Siberian lineages, as well as M segment of Siberian lineage are not available), the ML trees based on M (Fig. 2b) and L segments (Fig. 2c) supported 4 and 3 of these lineages. We further compared the amino acid sequences for each segment to evaluate lineage-specific amino acid substitutions. Notably, members of Middle-Eastern lineage had a clear signature amino acid pattern based on all three viral proteins. Some of these substitutions were in between biochemically different (i.e. between hydrophobic and hydrophilic amino acids) or between conformationally different amino acids (i.e., glycine or proline) suggesting that there may be also structural or functional differences between the TULV lineages.

**Fig. 3:**
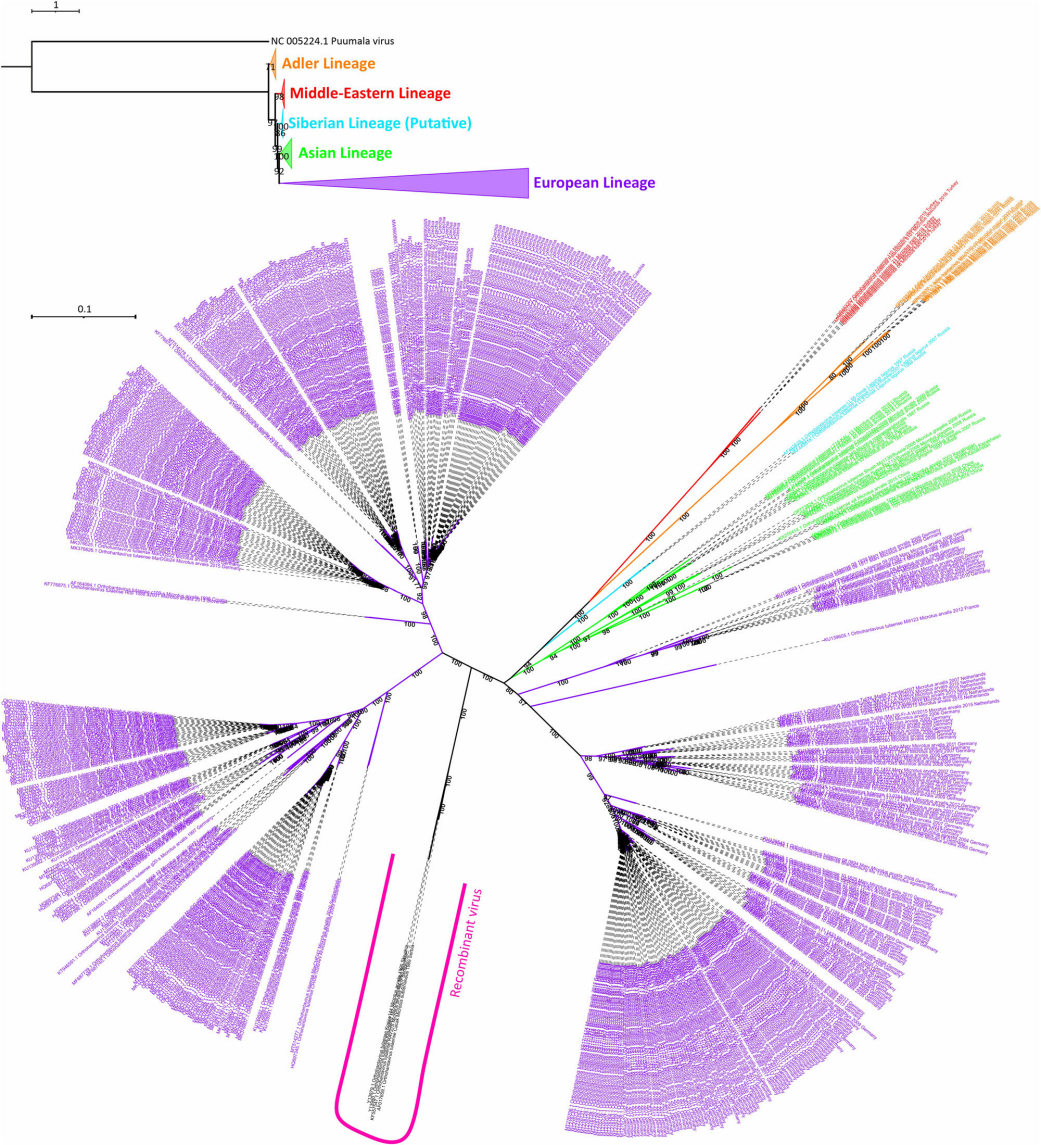
Illustration of ML trees, as both rooted (at the top) and unrooted (at the bottom), based on partial S segment dataset from TULV sequences. Rooted tree branches were collapsed according to each lineage and *O. puumalaense* reference sequence was used as an outgroup. TIM2 + F + R6 substitution model was used for rooted tree construction. In the unrooted tree, only TULV sequences were included. GTR + F + I + R4 substitution model was used during unrooted tree construction. The lineages colored as: Adler (orange), Middle-East (red), putative Siberian (cyan), Asian (green), and European (purple).

In order to confirm the tree topology with a dataset consisted of higher number of sequences, we included all available partial (≥ 500 nt long) S segment sequences to the analysis. This resulted as 565 sequences with 1302 nucleotide sites, and these sites had 328 constant and/or ambiguous constant sites (25.192% of all sites), 530 parsimony informative sites, and 837 distinct site patterns. The ML tree of S segment partial dataset supported lineage separation we observed based on CDS phylogeny, and provided higher bootstrap values (Fig. 3). Additionally, the ML tree of partial dataset provided two important aspect: (1) clearer separation of sub-clusters within each lineage; and (2) the lack of sampling and screening for other regions against heavy and repetative sampling in Europe, especially in central Europe.

### Phylogeographic reconstruction of *Orthohantavirus tulaense*

In order to further assess the geographic spread of TULV, we conducted Bayesian phylogeographic reconstruction in continuous space. First, we assessed the sufficiency of phylogenetic information for each segment’s dataset by likelihood mapping. We used 10,000 randomly chosen quartets to assess the phylogenetic information, which resulted as 87.4%, 95%, and 98.6% to be placed at the corners for S, M, and L segment, respectively (Supplemetary Fig. S3). The proportion of quartets placed in the center were 5.2%, 1.5%, and 0.7% for S, M, and L segment, respectively, suggesting that datasets provide enough phylogenetic information for further analysis. We evaluated the significance of phylogeographic information embedded into Bayesian trees by measuring association index (AI) and parsimony score (PS) which found to be significant (*P* = 0.0000) for all three segments based on both AI and PS.

**Fig. 4:**
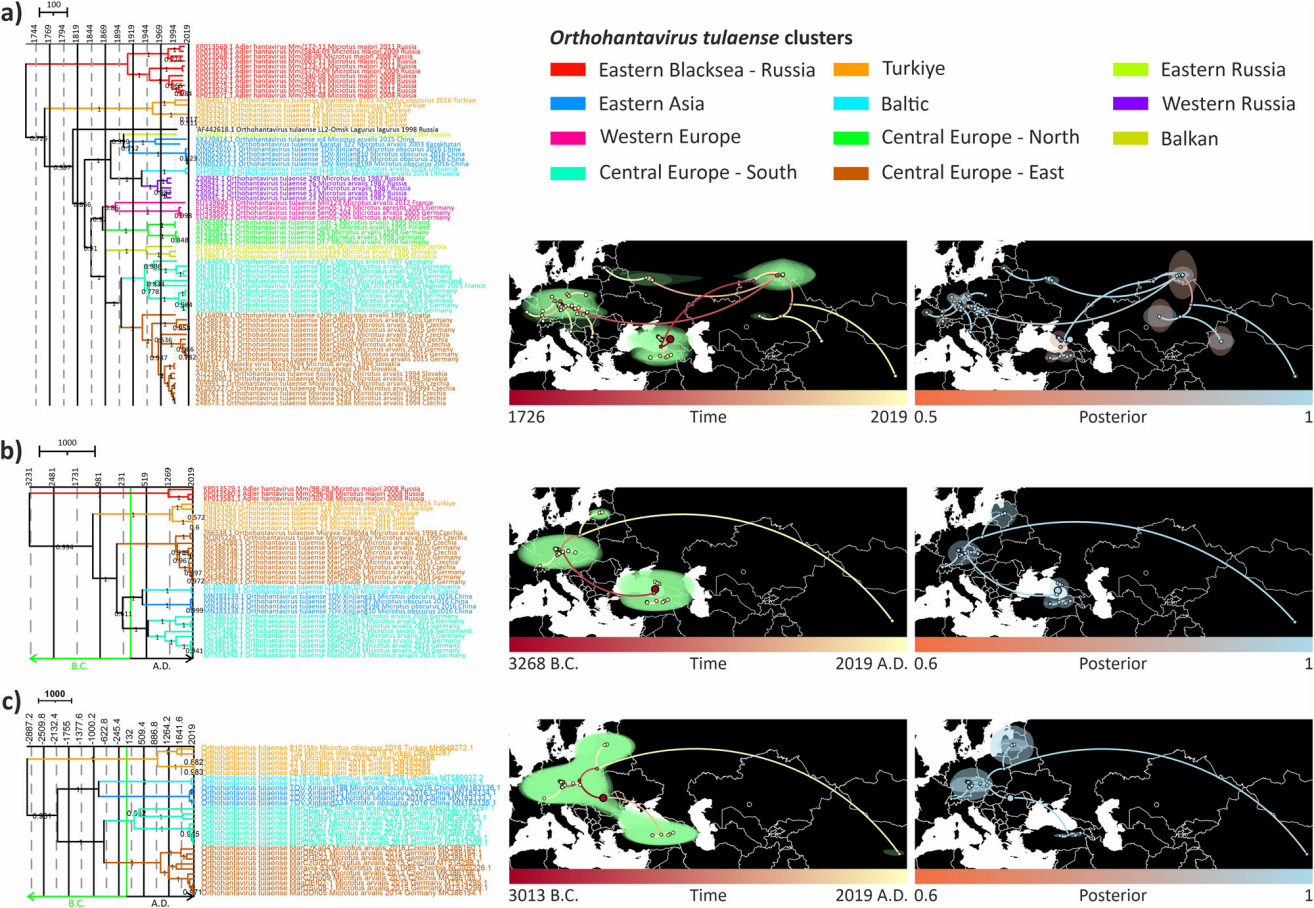
Phylogeographic reconstruction of TULV for each three segment with MCC trees (left), dispersal histories (center) and their posterior probabilities (right). Each segment’s trees were post-burn-in sampled to 1000 trees and fitted into MCC trees. Polygons show 80% highest posterior density (HPD) regions. Each node on the mapped MCC trees colored according to their estimated age from oldest (red) to younguest (yellow). **a** S segment, **b** M segment, and **c** L segment.

The tree topologies were largely congruent to ML-trees. For finer geographical resolution, the major lineages were further divided into 11 geographic clusters with one undefined cluster due to lack of data. These clusters were as follows: Eastern Black Sea—Russia (Adler lineage), Turkiye (Middle-Eastern lineage), Eastern Russia (Asian lineage), Eastern Asia (Asian lineage), Western Russia (Asian lineage), Baltic (Asian lineage), Balkan (European lineage), Western Europe (European lineage), Central Europe—North (European lineage), Central Europe—South (European lineage), Central Europe—East (European lineage) (Fig. 4a). One TULV sequence from *Lagurus lagurus* reported from Omsk region, Siberia, formed an outlier in S segment tree, hence we did not classify it under any cluster. Posterior probability distribution across S segment MCC tree was relatively high. However, some nodes such as at the node separating Turkiye cluster from rest of the Asian and European clusters, had a lower posterior probability (0.726). This may suggest that more divergent sequences from this or surrounding areas may be lacking.

The estimated evolutionary rates and times for the most recent common ancestors (tMRCAs) differed between the segments (Table 1, Fig. 4). For S segment the evolution rate and tMRCA were 1.257 × 10^-3^ substitutions/site/year and 1726 [95% HPD: 1562 – 1861], which correlates with a recent study^37^, whereas for M and L segments showed slower evolutionary rates [M segment = 7.019 × 10^-4^, L segment = 4.39 × 10^-4 ^s/s/y] and earlier estimates for tMRCAs [M segment = 3603 B.C., L segment = 2610 B.C.]. The low coefficient of variation estimates suggested that there were no large differences in the estimated evolutionary rates between branches (Table 1).

**Table. 1:** Evolution rates, tMRCA (time for most common recent ancestor), and coefficient of variation for each segment of TULV from phylogeographic reconstruction

	Evolution rate (substitution/site/year) [95% HPD interval]	tMRCA [95%HPD interval]	Coefficient of variation [95%HPD interval]
**S**	1.257 × 10^-3^ [6.984 × 10^-4^–1.943 × 10^-3^]	1726 [1562–1861]	0.229 [0.1008–0.3516]
**M**	7.019 × 10^-4^ [1.995 × 10^-7^–1.639 × 10^-3^]	3603 B.C. [9277 B.C.–1824 A.D.]	0.18 [0.0193–0.3188]
**L**	4.39 × 10^-4^ [5.6214 × 10^-7^–1.077 × 10^-3^]	2610 B.C. [11475 B.C.–1782 A.D.]	0.136 [0.0242–0.2514]

Estimated internal node locations in phylogeographic reconstruction were slightly different as compared to the previous study, most likely as a result of more sequences added from Turkiye and usage of only CDS in our analysis. Even though inference with CDS provided more phylogenetic information and allowed us to see real divergence for S segment, availability of CDS from critically important regions could have added more insight to our estimates and provided higher posterior probabilities. The tree topologies were congruent between segments (Fig. 4), except for the clades where no sequences are available for M and L segments. Posterior probabilities for both segments’ MCC trees were relatively higher than S segment since more genetic information embedded in these segments due to their length. tMRCAs were estimated as 3268 B.C. and 3013 B.C. for M and L segments, respectively (Fig. 4). Estimated rooting location for M segment was Eastern Black Sea region similar to S segments, but L segment was in Eastern Europe, most likely due to the lack of L segment sequences from Adler lineage. In the phylogeographic reconstruction, posterior probabilities of estimated internal nodes and root locations seemed much higher for M and L segment than S segment.

**Fig. 5:**
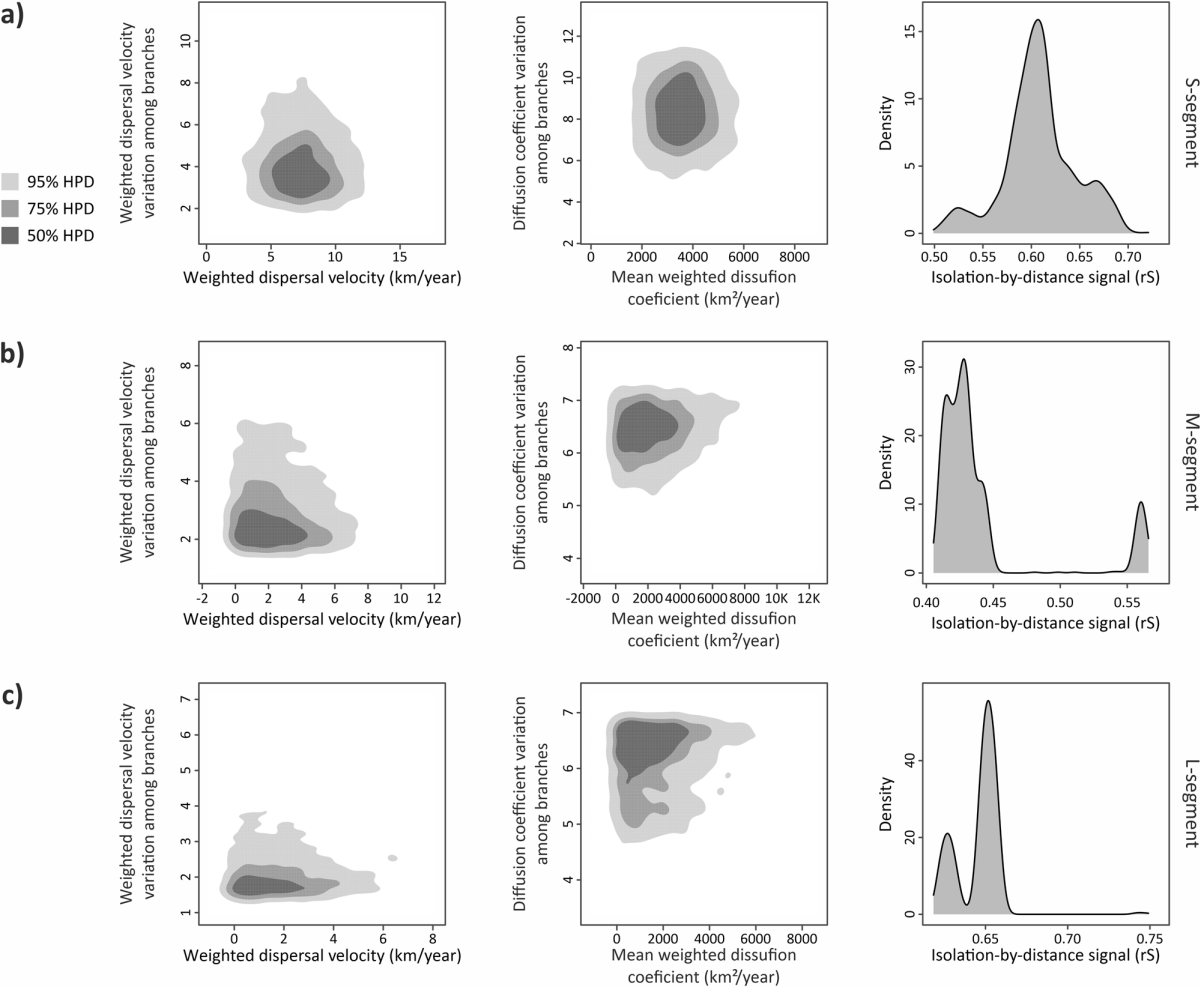
Dispersal statistics for each segment of TULV with 50%, 75%, and 95% HPD. On the left column, weighted dispersal velocity (km/year) rates against variation among each branch on the tree. On the central column, mean weigthed diffusion coefficient (km^2^/year) against variation among each branch. On the right column, IBD signals for the level of phylogeographic structure with rS signals. **a** S segment, **b** M segment’ and **c** L segment.

We further calculated global dispersal statistics for each segment (Fig. 5). Weighted dispersal velocity for each branch in the trees were 7.3 km/year, 2.05 km/year, and 1.411 km/year on average for S (Fig. 5a), M (Fig. 5b), and L segments (Fig. 5c), respectively. The diffusion coefficient rates for virus spread to a given space in terms of surface area had mean values as 3528.97 km^2^/year, 2014.9 km^2^/year, and 1457.9 km^2^/year for each segment from S to L segments. Variation of dispersal statistics among branches for both dispersal velocity and diffusion coefficient were slightly higher for S segment to other segments. The lower estimated rates of dispersal statistics across segments may be as a result of lacking genetically divergent sequences in M and especially L segment datasets. Additionally, we calculated isolation-by-distance (IBD) signal by using the Spearman correlation (rS). S and L segments gave strong signals with 0.61 and 0.65 which means these segments give phylogeographic structure. However, IBD signal for M segment was lower than other two with the rate of 0.43.

**Fig. 6:**
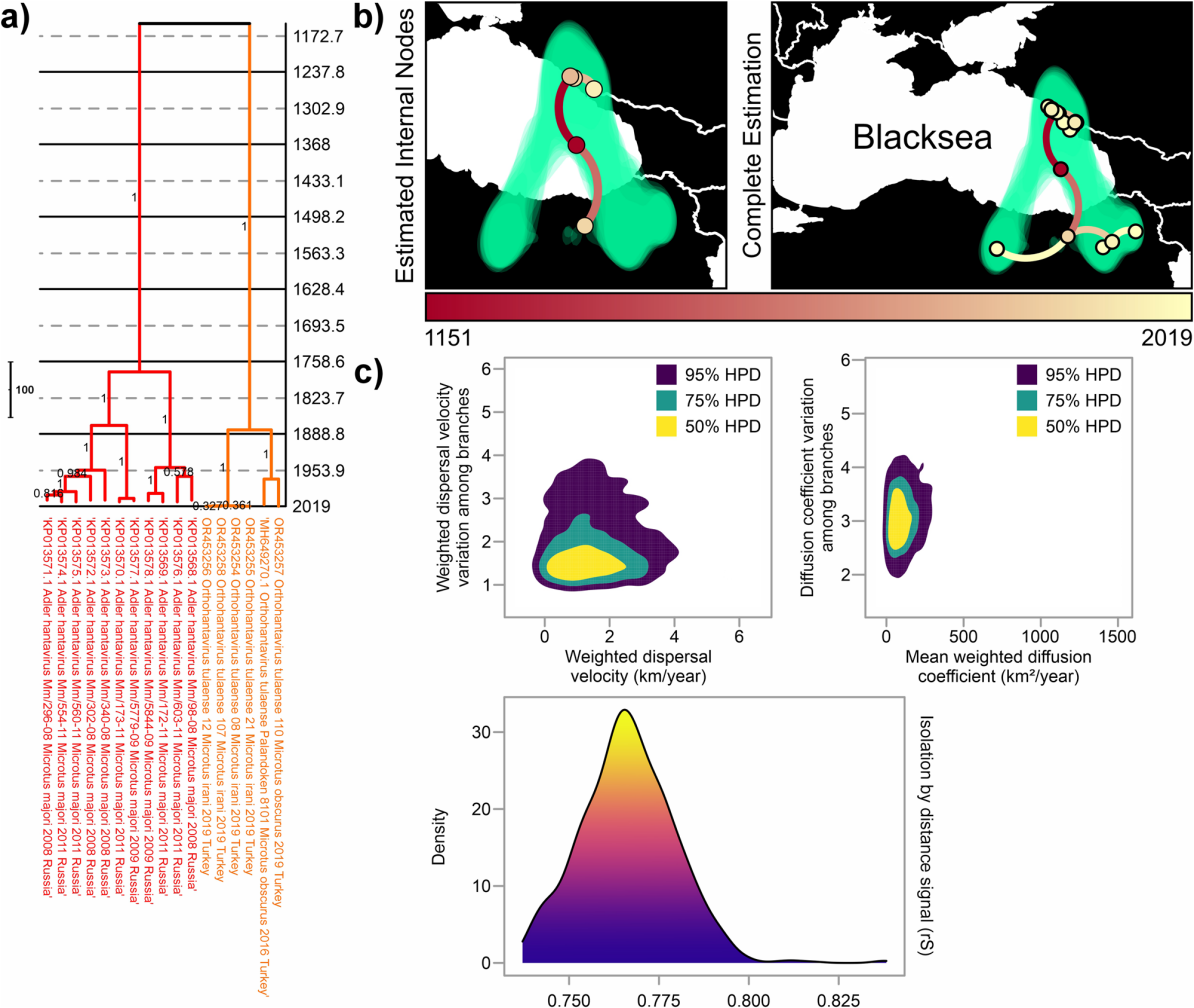
Phylogeographic reconstruction and dispersal estimates for TULV Adler and Middle-Eastern lineages from their S segments. **a** Time-scaled MCC tree is colored according to lineage colors described previously. **b** Estimation of phylogeography of these lineages. On the left, estimated locations of possible ancestors, named as internal nodes, of these lineages. On the right, complete analysis that includes both estimated internal nodes and locations where TULV positives were observed. **c** Calculation of dispersal statistics was conducted for regional spread of these two lineages.

Finally, we conducted phylogeographic reconstruction of S segment focusing on Black Sea region in order to further resolve the distribution pattern in the root location of TULV (Adler and Middle-Eastern lineages) (Fig. 6a). In contrast to entire S segment dataset for all lineages, this regional estimation suggested older common ancestor for Adler and Middle-Eastern lineages dating to year 1151 [95% HPD: 194.77 B.C.–1951.65 A.D.] (Fig. 6a, b). Estimated internal nodes for each lineage were separate from one another (Fig. 6b), suggesting independent evolution of these lineages in northern and southern coasts of Eastern Black Sea. Dispersal speed estimations for these two lineages in Black Sea region were 1.2751 km/year [95% HPD: 0.02457 km/year–3.1162 km/year] for branch dispersal velocity and 91.75 km^2^/year [95% HPD: 0.5067 km^2^/year–249.8191 km^2^/year] for diffusion coefficient (Fig. 6c). Both lineages also had strong IBD signal with rate of 0.76 (Fig. 6c).

### Comparative and regional inference of *Orthohantavirus tulaense* evolution rates

Altogether, the analyses above suggested a considerable difference between the evolutionary rate and dispersal speed estimates of S segment based on the complete dataset and the dataset consisting Middle-Eastern and Adler lineages only. Therefore, in order to reduce the potential sampling bias due to large scale sampling in Europe, we further analyzed a dataset that contained only the TULV strains of which the sequences of all three segments were available. According to this analysis, estimated evolution rates were approximately equal; 7.603 × 10^-4 ^s/s/y, 7.0101 × 10^-4 ^s/s/y, and 5.0935 × 10^-4 ^s/s/y for S, M, and L segments (Table 2), respectively. This suggests that short term evolutionary rate represented by heavily sampled European lineage may be faster than long term evolutionary rate, therefore introducing a bias in the estimates based on the complete S-segment dataset.

**Table. 2:** Evolution rates and tMRCA(s) of comparative and regional evolutionary inference for TULV

Dataset	Evolution rates [95% HPD Interval]	tMRCA [95% HPD Interval]	Molecular clock type
S segment (Unbiased dataset)	7.603 × 10^-4^ [7.7063 × 10^-6^–1.4975 × 10^-3^]	1230.345 [113.5948–1925.3471]	Uncorrelated relaxed
M segment (Unbiased dataset)	7.0101 × 10^-4^ [2.6877 × 10^-6^–1.6709 × 10^-3^]	1286.681 B.C. [5136.9899 B.C.–1901.2288 A.D]	Uncorrelated relaxed
L segment (Unbiased dataset)	5.0935 × 10^-4^ [4.29 × 10^-7^–1.2198 × 10^-3^]	2510 B.C [8040.4515 B.C–1791.0258 A.D]	Uncorrelated relaxed
S segment: Adler lineage	1.16×10^-3^ [3.4542×10^-4^ – 1.9943 × 10^-3^]	1887.885 [1768.6411–1964.8462]	Fixed local clock
S segment: Middle-Eastern Lineage	5.476×10^-4^ [5.0517×10^-5^–1.264 × 10^-3^]	1796.449 [1415.3756–1997.796]	Fixed local clock
S segment: whole datasets	7.8845 × 10^-4^ [3.4159 × 10^-4^–1.2418 × 10^-3^]	1536.583 [1177.0346–1797.5094]	Fixed local clock
S segment: Adler lineage excluded	1.143 × 10^-3^ [1.8741 × 10^-4^–1.8586 × 10^-3^]	1745.113 [1547.23–1900.11]	Uncorrelated relaxed

Notably, this dataset did not include Adler lineage, since L segment sequences were not available for this lineage. To address the role of Adler lineage in the evolutionary rate estimates, we employed fixed local molecular clock model for S segment dataset, assuming that this lineage does not evolve according to global evolutionary rate. This analysis suggested that Adler lineage has evolved at faster rate than rest of the lineages. Adler lineage had estimated evolutionary rate of 1.16 × 10^-3 ^s/s/y, while the entire dataset and Middle Eastern lineages had estimated rates of 7.884 × 10^-4 ^s/s/y and 5.476 × 10^-4 ^s/s/y (Table 2), respectively.

We, finally, used same dataset but excluded Adler lineage to test the changes of evolution rate and the significance of this lineage. The estimated evolution rate was 1.143 × 10^-3^ with tMRCA of 1745.113.

### Host switching estimates of *Orthohantavirus tulaense*

**Fig. 7:**
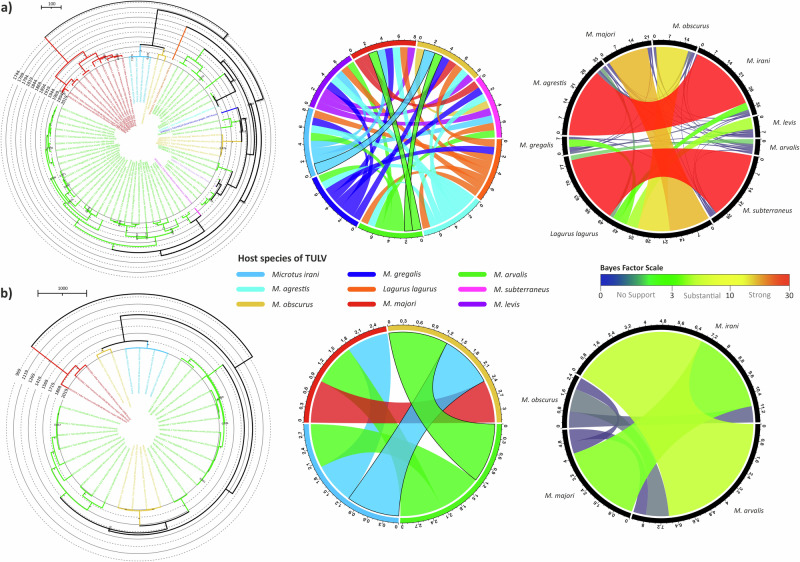
Host switching inference for TULV. The left column illustrates the time-scaled MCC trees from host inference analysis. MCC tree branches are colored according to TULV host species. Central column shows Bayesian signals for each pair of rodent species to estimate the potential jumps, and ones above the cut-off (1.0) marked with straight black lines. Right panel gives the confidence of each hypothesis for host jumps of TULV according to traditional scaling of bayes factors with rainbow coloring. **a** S segment, and **b** M segment.

In order to infer the role of host-switching events in the TULV evolution, we also estimated host jump rates. L segment was excluded from this analysis, since this segment is available only from three rodent species. Our analysis included sequences from nine and four different rodent species of which TULV for S and M segment sequences were available, respectively. We, again, evaluated the significance of host species information embedded into TULV Bayesian trees by measuring AI and PS, which both were significant (*P* = 0.0000) for both segments. The MCC trees for both segments showed clear host-dependent sub-clustering in given geographic clusters (Fig. 7). With host switching signal cut-off 1.0, the jump events seemed to occur in the given order: *M. arvalis* to *M. obscurus* and to *M. irani* (Fig. 7). However, the Bayes factors suggested that there was no evidence to support this hypothesis (Fig. 7). This may be due to heavy sampling for *M. arvalis* against low sampling for other species. Strikingly, the signal for *M. arvalis* and *M. irani* was 0.921 reflecting there were no strong signal for any switching event between these two, and this hypothesis had substantial evidence according to Bayes factor scale. This suggests there might be other rodent species involved in shedding the virus which hasn’t been identified yet.

## Discussion

Understanding the diversity of orthohantaviruses, their geographic distribution, and their adaptive evolutionary dynamics is of importance for understanding their zoonotic capability. One approach to gain such information is performing wildlife surveillance to obtain molecular data. We performed a cross-sectional study to search for orthohantaviruses from eastern Turkiye, sequenced new divergent strains of *Orthohantavirus tulaense* (TULV) and characterized TULV global lineage distribution, reconstructed phylogeography along with estimating dispersal statistics in both global- and regional-scale, and inferred host switching events with currently available complete coding sequence (CDS) data.

Wild rodents positive for orthohantaviruses, as well as diagnosis of human infections and outbreaks have been reported from Turkiye previously. The human infections and outbreak regions have mostly been observed in northern parts of Turkiye, and responsible virus species were *O. dobravaense* (DOBV) and *O. puumalaense* (PUUV)^38–42^. Also, studies have shown wild rodents positive for some orthohantaviruses, such as DOBV or TULV, in northern and eastern part of Turkiye^21,43,44^. A regional overlap is evident for TULV, DOBV, and PUUV when considering both human and rodent findings. The active and continuous wild rodent surveillance, detection of novel viruses, and understanding viral dynamics in every aspect for orthohantavirus evolution are essential for early detection and prevention in this region.

**Fig. 8:**
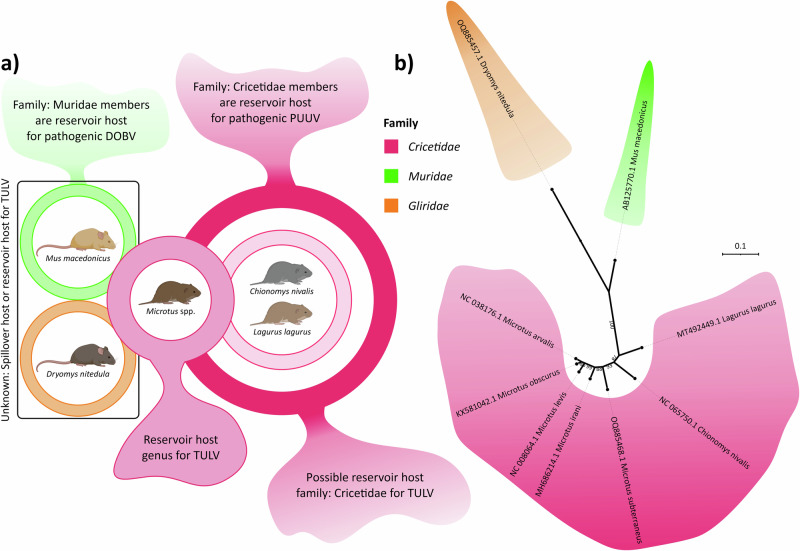
Diversity of host species which were reported to carry TULV, and how these host species are distant from one another in family level as three rodent families emerges: *Cricetidae* (pink; as main reservoir host family), *Muridae* (green) and *Gliridae* (orange). **a** Panel shows rodent species reported to have TULV, family relationships of these reported species and pathogenic orthohantaviruses they carry to example for potential host overlaps between different *Orthohantavirus* species. **b** ML phylogenetic tree illustrates distant genetic relationship of these three rodent families based on complete *cyt-b* genes to give perspective on possible molecular and phenotypic divergence of TULV in the sense of host species specificity.

The traditional paradigm for orthohantavirus co-evolution with their host species suggested that one virus-one reservoir host species pairings led the speciation of orthohantaviruses^14,45,46^. This paradigm was challenged by studies indicating host-switching events, higher evolutionary rates, and novel *Hantavirus* (other members of *Hantaviridae*) reports from a variety of animals^47–49^. Indeed, TULV seems to have a wider host range than most other orthohantaviruses including DOBV and PUUV. The recent studies from Kazakhstan and Iran reported TULV positive *Dryomys nitedula* rodents^20,22^, which is a different rodent family (*Gliridae*) from the previously suggested TULV primary reservoir host family *Cricetidae* (Fig. 8a). In addition, data obtained from our study also showed potential spillover to *Mus macedonicus*, which is again different family (*Muridae*) (Fig. 8), suggesting that TULV can infect genetically divergent rodents of at least three families (*Cricetidae*, *Muridae*, and *Gliridae*).

Previous research has suggested a number of TULV lineages and cluster characterizations, mainly focusing on specific regions and with limited data^22,50,51^. Here we combined all the genomic data that has accumulated from different geographical locations and diversity of host species, for better phylogenetic resolution. We characterized 5 lineages from ML trees, further divided into 11 clusters. Both lineage separation and clusters showed strong phylogeographic structure, consistent with the hypothesis that geographic barriers may play role on genetic diversity and clade separation of orthohantaviruses^52,53^.However, the long branch lengths and low support for some of the earliest node in the trees suggest that there may be unsampled diverse TULV strains, especially in the Black Sea, Middle-East and Siberia.

Conspicuously, the observed number of Middle-Eastern lineage-specific amino acid substitutions, most of which also change the biochemical properties of the given amino acid, suggests that host switch events of TULV may be followed by diversifying adaptive evolution. At finer scale, diversifying adaptive evolution has been previously shown in the N-terminus of M segment between two different TULV clusters circulating in two different *Microtus arvalis* genetic clusters in Central Europe^51,54,55^. Even though we focused only on the lineage-specific amino acid pattern of Middle-Eastern lineage (TULV sequences from Turkiye) in our study, these results highlight the need for complete genome sequences in order to understand the drivers for speciation among orthohantaviruses.

A recent TULV phylogeography study with partial S segment dataset proposed three major introductions from the rooting location which was predicted to be the northeast Black Sea region^37^. These introductions were to Europe, Asia, and Middle East. Our phylogeographic reconstruction for S segment with CDS dataset seemed to be in correlation with these phylogeographic introductions and estimating potential location of common ancestor. On the other hand, M segment estimations for internal node locations showed deviation to S segment. This was mostly caused by lack of data from Siberian and Asian lineages. L segment dataset did not include sequences from Adler lineage since there were no available CDS for TULV from this lineage for this segment, hence the estimation of common ancestor location was different. Interestingly, regional phylogeographic reconstruction for eastern Black Sea suggested independent internal node estimations for Adler and Middle-Eastern lineages which dated back much older than the entire dataset. This suggests that these two lineages have evolved separately and might be the closest relatives to the common ancestor of all currently known Tula viruses. This also highlights the importance of conducting TULV study in Georgia and Armenia where seemed to have some of the key areas for TULV evolution. Another recent study on DOBV phylogenetic characterization with phylogeographic reconstruction in discreet space revealed that lineage separation was determined by reservoir host species followed by geographic clustering^56^. For TULV, this phylogenetic pattern was other way around, meaning lineage forming and clustering determined by geography followed by host-dependent subclustering. This suggests that evolutionary patterns of different orthohantavirus species may differ from one another. What’s more, this study on DOBV, a *Murinae*-borne *orthohantavirus*, may be dating back to 1818 according to phylogeographic estimates^56^. However, our study suggested TULV, an *Arvicolinae*-borne *ortohantavirus*, estimated as much older than DOBV.

Recent development on dispersal calculation methods enables understanding virus diffusion dynamics in a given space or population. Isolation-by-distance (IBD) is one of these methods and can be used together with dispersal velocity and diffusion coefficiency to understand how genetically isolated viruses are and how fast they have capability to spread^36^. Previously, the rates for diffusion coefficiency and IBD were estimated as 1 km^2^/year and 0.82 for PUUV in Belgium, respectively, and 399 km^2^/year and 0.52 for TULV in central Europe. Our estimates for global TULV diffusion dynamics (3528.97 km^2^/year and 0.61) align with these previous estimates, considering that the previous study used sub-clades within virus species compared to global TULV diversity used in our study. However, there are some intriguing differences between TULV and PUUV, namely, PUUV appears to evolve at lower rate and disperse at lower velocity than TULV. Speculatively, this might be linked to much wider host range of TULV (Fig. 8a), as compared to PUUV, which have only one host species, the bank vole (*Myodes glareolus*). Additionally, even though region-focused calculations for Black Sea region showed significant drop in the dispersal velocity in comparison to global-scale, diffusion coefficient rate for two lineages, Adler and Middle-Eastern, in this area remained high with rate of 91.75 km^2^/year. Since this area seems to have TULV in quite a diverse species of reservoir hosts, this level of diffusion coefficient may provide some evidence to the effects of wide host range.

The evolutionary rates between 10^-2^ and 10^-4^ substitution/site/year have been previously estimated for orthohantaviruses^49,57^. Our results for TULV were consistent with these earlier estimations. Notably however, the evolutionary rate estimations for M and L segments were lower than that for S segment, resulting as earlier tMRCAs, albeit with relatively wide confidence interval range. Given the wide geographic distribution (spanning from Europe to Eastern Asia), the tMRCA for S segment is most likely underestimated (as also discussed in previous study^37^). Likewise, the related global dispersal velocity estimate 7.3 km/year for S segment seems unrealitically high for a rodent-borne virus.

To gain more insight into this discrepancy, we downsampled the intensively studied European lineage by taking into account only the TULV strains, of which sequences from all three segments were available, as well as performed additional analyses focusing on the divergent lineages from the Black Sea region. These analyses suggested lower evolutionary rate estimates for S segment, comparable for those of TULV M and L segments, as well as those previously estimated PUUV S segment evolutionary rate. Further, Adler lineage seems to evolve faster than the other lineages. Altogether, these analyses suggest that the S segment evolutionary rate based on the complete dataset may be overestimated both due to biased dataset (overweighting faster short-term evolution in expense of slower long-term evolution), as well as due to potential episodic adaptive evolution, as also suggested by lineage-specific signature amino acid patterns. Overall, eastern Black Sea region came forth for its importance on high diversity and its close relation to the common ancestor virus from this region.

While *Microtus arvalis* has been thought to be the primary reservoir host of TULV, recent studies and our findings here have shown that the host range of TULV might be much wider than previously thought (Fig. 8a). Although our study gives insight into the role of host-switch events in TULV evolution, it is evident that the current data is still insufficient to capture the complete view of TULV host species diversity. Our estimates did not show any support for direct host-switch event between the traditional *M. arvalis* reservoir host and newly identified host *M. irani* suggesting a role for yet unidentified host species for TULV. For example, several *Microtus* species such as *Microtus elbeyli* and *Microtus guentheri*, are found in Turkiye^58,59^ and some of them haven’t been screened specifically for TULV.

TULV is a rodent-borne orthohantavirus with unknown pathogenicity, and understanding its host range, evolutionary dynamics and spatio-temporal characteristics are important. Our results showed TULV circulation in *M. irani, M. obscurus* and in their close relative *C. nivalis*. The recent detections of TULV in different rodent species provides a clearer picture on host range and diversity of orthohantaviruses. Eastern Black Sea region and related lineages from this area seemed to have unique traits in terms of their evolution and diversity. This region may be one of the key areas for understanding the origin of TULV and perhaps much more. The results warrant further studies from a wider range of rodent species especially in the understudied regions, such as Georgia or Armenia, where divergent TULV clades most likely circulate.

## Supplementary information

Erdin_etal_Supplementary_Material_Datasets

Supplementary Information

## Data Availability

Data is provided within the manuscript and/or supplementary information files. Sequence data provided within the manuscript submitted to NCBI GenBank database and accession numbers will be available once the study is published.
