# Supplementary material for: Detection of divergent Orthohantavirus tulaense provides insight into wide host range and viral evolutionary patterns
Source: Npj Viruses. 2024 Dec 4;2:62. doi: 10.1038/s44298-024-00072-y (PMC11721384; doi:10.1038/s44298-024-00072-y)
Supplement: Supplementary file 2 — Supplementary Information [file 44298_2024_72_MOESM2_ESM.docx]

**Supporting information**

**
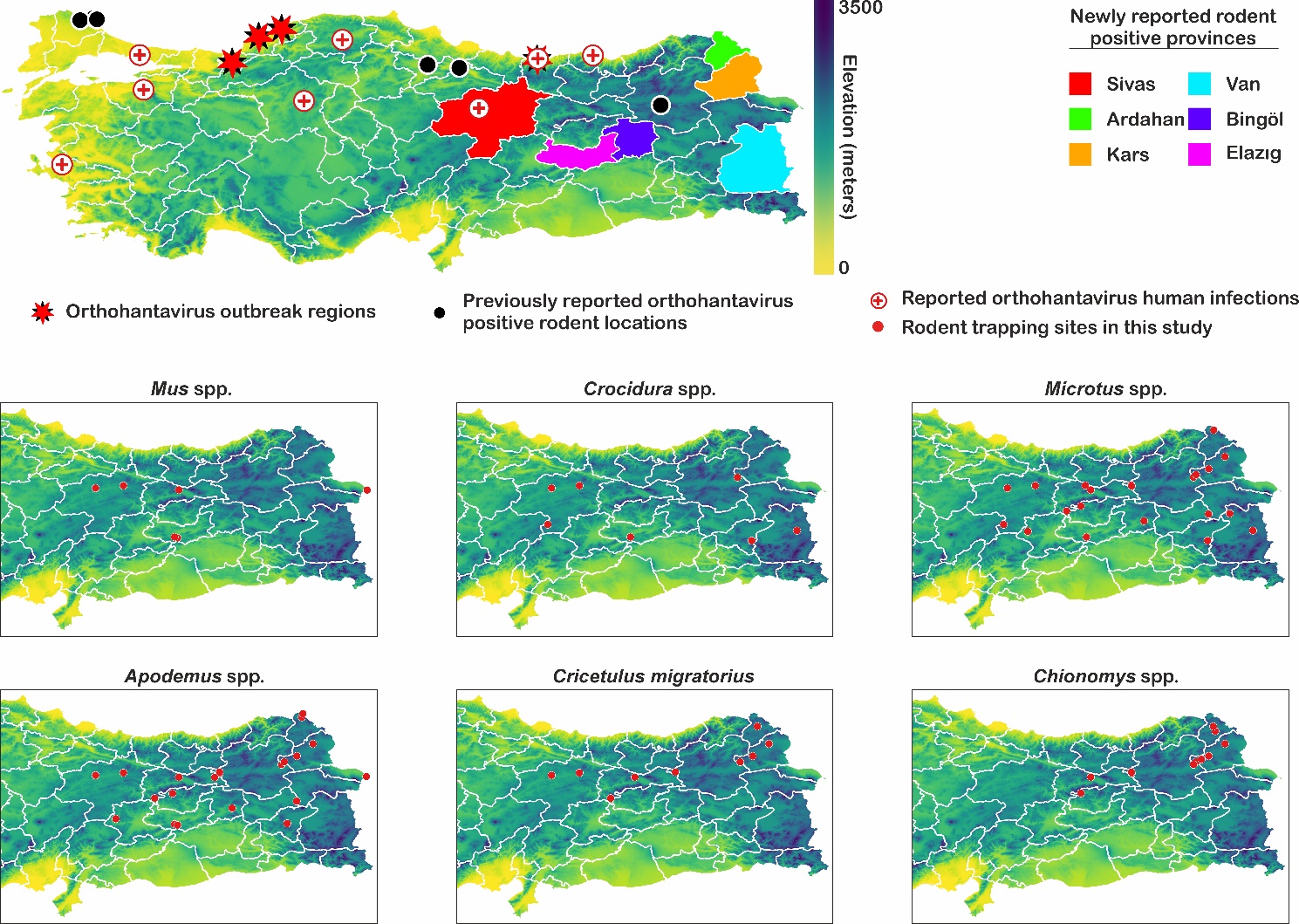
**

**Supplementary Figure S1.** Maps showing sampling regions for each targeted rodent species during field studies.

**Supplemetary Table S1.** Numbers of rodents as genera were captured in our field studies, and percentage of TULV positives against rodent genus collected from the entire region.

| **Provinces** | **Rodent genera** | | | | | | **Total** |
| --- | --- | --- | --- | --- | --- | --- | --- |
|  | *Apodemus* | *Microtus* | *Mus* | *Chionomys* | *Cricetulus* | *Crocidura* |  |
| Sivas | 7 | 20 | 4 | - | 5 | 7 | **43** |
| Erzincan | 6 | 10 | 1 | 2 | 2 | - | **21** |
| Erzurum | 8 | 21 | - | 22 | 15 | 1 | **67** |
| Kars | 11 | 5 | - | 9 | 6 | - | **31** |
| Ardahan | 6 | 5 | - | 7 | 3 | - | **21** |
| Iğdır | 1 | - | 11 | - | - | - | **12** |
| Malatya | 13 | 1 | - | - | - | - | **14** |
| Elazığ | 2 | 12 | 11 | - | - | 9 | **34** |
| Tunceli | 8 | 5 | - | 1 | - | - | **14** |
| Bingöl | 2 | 7 | - | - | - | - | **9** |
| Bitlis | 1 | 4 | - | - | - | 2 | **7** |
| Van | - | 14 | - | - | - | 2 | **16** |
| Muş | 1 | 3 | - | - | - | - | **4** |
| **Total** | **66** | **107** | **27** | **41** | **31** | **21** | **193** |
| **Detection of TULV against entire collection (%)** | **0** | **11.21** | **4.76** | **4.88** | **0** | **0** |  |

**
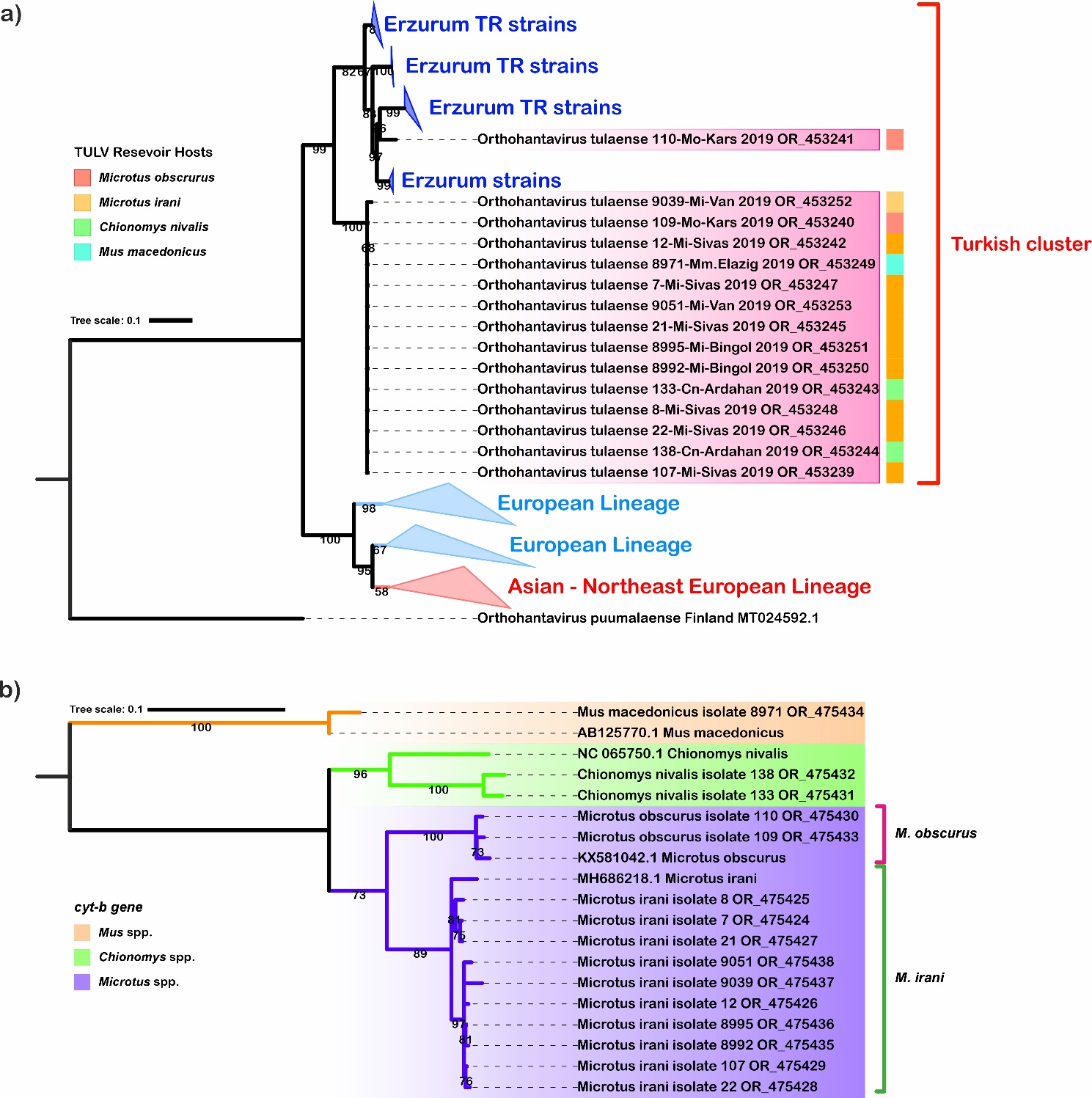
**

**Supplementary Figure S2.** (A) ML tree of partial L-segment sequences with TIM2+F+I+R3 substitution model and 1000 ultrafast bootstrap replicates. Partial L-segment sequences were clustered with previously published partial sequences from the same region of Turkey. This also confirms that PCR positive rodents were TULV. Defined clusters and lineages were preliminary and further characterization was performed with CDS. (B) Molecular species identification of positive reservoir hosts in ML tree with TVM+F+I+G4 and 1000 ultrafast bootstrap replicates.


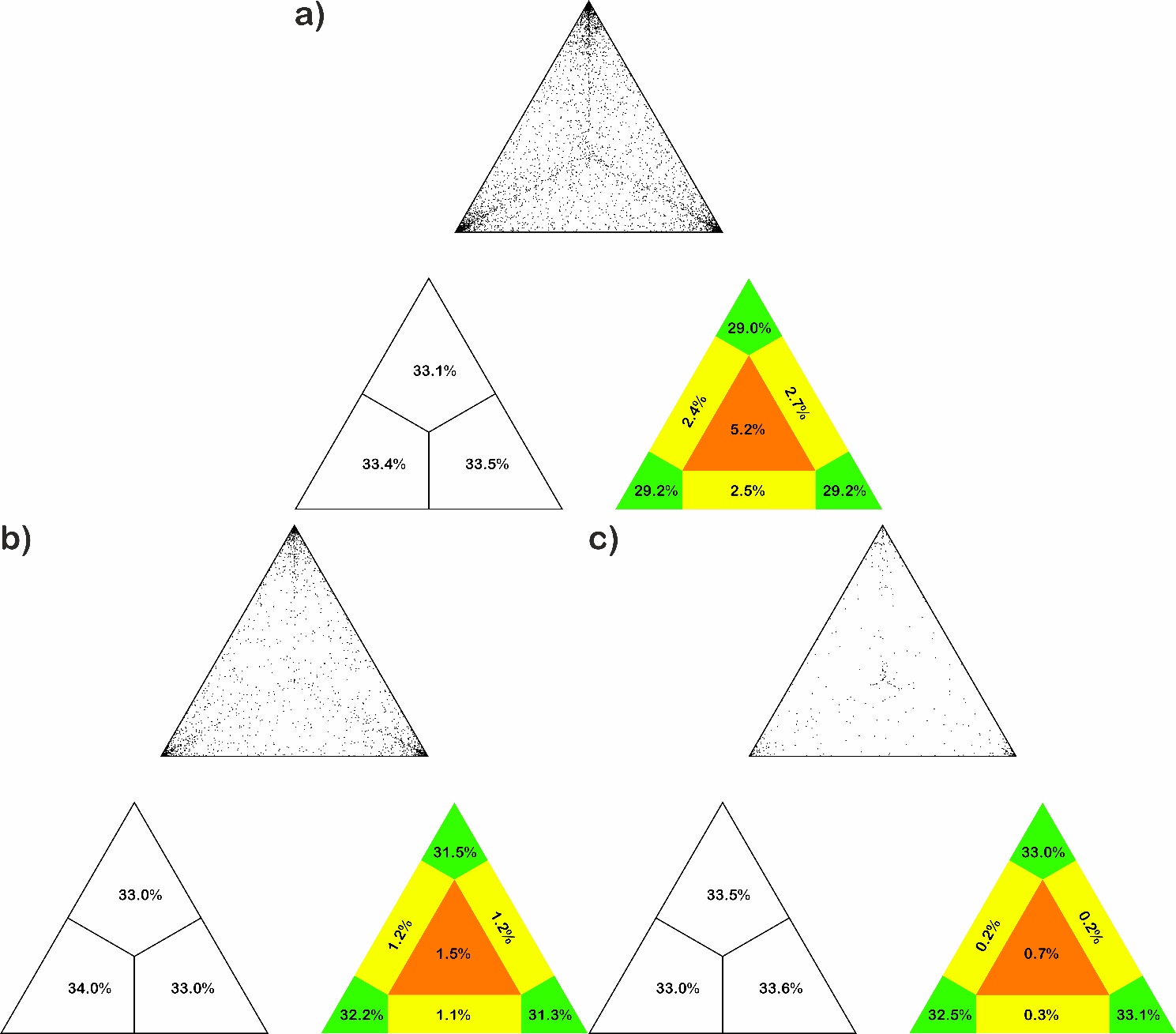


**Supplemetary Figure S3:** Likelihood mapping of each three segment. (**a**) S-segment, (**b**) M-segment, and (**c**) L-segment.
